# The Contribution of Common and Specific Therapeutic Factors to Mindfulness-Based Intervention Outcomes

**DOI:** 10.3389/fpsyg.2020.603394

**Published:** 2021-01-14

**Authors:** Nicholas K. Canby, Kristina Eichel, Jared Lindahl, Sathiarith Chau, James Cordova, Willoughby B. Britton

**Affiliations:** ^1^Department of Psychology, Clark University, Worcester, MA, United States; ^2^Department of Psychiatry and Human Behavior, Warren Alpert Medical School, Brown University, Providence, RI, United States; ^3^Department of Religious Studies, Brown University, Providence, RI, United States

**Keywords:** therapeutic alliance, group cohesion, common factors across psychotherapies, mindfulness, meditation, MBSR/MBCT, mindfulness-based cognitive therapy

## Abstract

While Mindfulness-Based Interventions (MBIs) have been shown to be effective for a range of patient populations and outcomes, a question remains as to the role of common therapeutic factors, as opposed to the specific effects of mindfulness practice, in contributing to patient improvements. This project used a mixed-method design to investigate the contribution of specific (mindfulness practice-related) and common (instructor and group related) therapeutic factors to client improvements within an MBI. Participants with mild-severe depression (*N* = 104; 73% female, *M* age = 40.28) participated in an 8-week MBI. Specific therapeutic factors (formal out-of-class meditation minutes and informal mindfulness practice frequency) and social common factors (instructor and group ratings) were entered into multilevel growth curve models to predict changes in depression, anxiety, stress, and mindfulness at six timepoints from baseline to 3-month follow-up. Qualitative interviews with participants provided rich descriptions of how instructor and group related factors played a role in therapeutic trajectories. Findings indicated that instructor ratings predicted changes in depression and stress, group ratings predicted changes in stress and self-reported mindfulness, and formal meditation predicted changes in anxiety and stress, while informal mindfulness practice did not predict client improvements. Social common factors were stronger predictors of improvements in depression, stress, and self-reported mindfulness than specific mindfulness practice-related factors. Qualitative data supported the importance of relationships with instructor and group members, involving bonding, expressing feelings, and instilling hope. Our findings dispel the myth that MBI outcomes are exclusively the result of mindfulness meditation practice, and suggest that social common factors may account for much of the effects of these interventions. Further research on meditation should take into consideration the effects of social context and other common therapeutic factors.

## Introduction

Mindfulness-based interventions (MBIs) are widely used to address a variety of conditions, including stress, anxiety, depression, and well-being (Grossman et al., 2004; Eberth and Sedlmeier, 2012). The two most common MBIs, Mindfulness-Based Stress Reduction (MBSR; Kabat-Zinn, 1990) and Mindfulness-Based Cognitive Therapy (MBCT; Segal et al., 2002), are structured 8-week group interventions that meet once a week for 2.5–3 h and are led by a trained instructor. Mindfulness meditation plays a central role, and is typically practiced for more than an hour of each class session as well as for an additional hour of home practice each day between sessions, and following treatment (Segal et al., 2002; Santorelli et al., 2017).

While it is widely assumed that the practice of mindfulness meditation, both within and outside the treatment sessions is “the component critical to effectiveness” (Williams et al., 2014, p. 276), this assumption is not strongly supported by current research. Correlation studies that assess the relationship between meditation practice amount and beneficial outcomes have yielded mixed results. Parsons et al. (2017) found that 75% of MBSR and MBCT studies showed no significant relationship between practice amount and outcomes.

Mindfulness-based interventions have been repeatedly found to be largely equivalent to other established treatments (Goyal et al., 2014; Goldberg et al., 2018). On one hand, this equivalence could mean that common therapeutic factors rather than treatment approach or treatment-specific components (i.e., meditation) are responsible for efficacy. On the other hand, treatment equivalency could also mean that different treatments achieve roughly equal benefits through different mechanisms, but that these treatment-specific components still matter (Rosenkranz et al., 2019). A recent systematic review of the literature on mindfulness meditation concluded that the contribution of common factors to MBIs, such as group and instructor support, is an important avenue for further research (Wielgosz et al., 2019).

Only a few studies have attempted to tease out these two possibilities empirically by creating a control condition that was matched to MBIs on all components except meditation practice. Using an innovative dismantling design, Williams et al. (2014) found that MBCT with and without meditation were equally efficacious, suggesting that other components of MBCT besides meditation practice may underlie its efficacy. The active control condition in the study by Williams et al. (2014) did not replace the time that participants in the MBCT condition spent meditating, thus achieving the same outcomes with a greatly reduced time commitment. Similarly, an active control group created to match the structure of MBSR but without mindfulness practice or instruction was equivalent to MBSR for psychological distress, stress response, and anxiety (MacCoon et al., 2012; Rosenkranz et al., 2013), as well as depression relapse, symptom reduction, and life satisfaction (Shallcross et al., 2015), differing only on post-stress inflammatory response and reductions in thermal pain ratings, or in temporal patterns at follow-up.

Theoretically, mindfulness interventions are developed on the premise that mindfulness meditation leads to greater mindfulness skills, which subsequently result in symptom reduction (e.g., Kabat-Zinn, 1990; Baer et al., 2008). However, there have been inconsistent findings regarding the relationship between mindfulness meditation practice and resulting increases in self-reported mindfulness. Self-reported mindfulness by definition is believed to be increased by mindfulness meditation practice (Baer et al., 2008) and has been found to mediate the effects of MBSR on outcomes (Bergen-Cico and Cheon, 2013). However, a systematic review found that both MBIs and active control groups lacking meditation resulted in improvements in self-reported mindfulness, with no significant differences between them (Visted et al., 2014). One study investigating this question found that both out-of-class meditation and therapeutic alliance predicted improvements in self-reported mindfulness (Bowen and Kurz, 2012), suggesting that mindfulness skills may result from common therapeutic factors as well as meditation.

The common factors perspective (Frank and Frank, 1993; Laska et al., 2014; Wampold and Imel, 2015) theorizes that most of the variance in psychotherapy outcomes is not the result of specific techniques but of contextual factors which exist in any “bona-fide” therapeutic approach, such as positive relational bonds, agreement on tasks and goals, hope/expectations for improvement, group dynamics, and a safe environment conducive to healing. While only a few studies have looked at the contribution of common factors to MBI outcomes, a comparison between specific and common factors across multiple clinical outcomes has not previously been conducted.

The common factors perspective has a long history of theory and debate in psychotherapy research. Rosenzweig (1936), p. 412, first speculated that “therapeutic result is not a reliable guide to the validity of theory” and originated the “Dodo bird verdict,” which theorized that all therapies may be equally effective. Common factors theory (continued by Laska et al., 2014) argues that therapy outcomes may reflect elements that are common to all “bona fide” therapies rather than the mechanisms of change supported by distinct theories. Large meta-analyses comparing the outcomes of different forms of therapy have found support for the dodo bird verdict, with findings both in general (e.g., Smith and Glass, 1977; Wampold et al., 1997; Luborsky et al., 2002) and for specific disorders (e.g., Leichsenring and Leibing, 2003; Benish et al., 2008).

To account for treatment equivalence, researchers have focused on identifying the common factors that exist within and across different therapeutic modalities. Some of the most frequently studied common factor variables in a group setting are therapeutic alliance, which includes both working and bonding dimensions with the therapist and with other group members (Bordin, 1979; Johnson et al., 2005; Norcross and Wampold, 2011; Laska et al., 2014), group dynamics such as group cohesion, group instillation of hope, secure emotional expression, interpersonal learning, and social impact (Yalom, 2005; Macnair-Semands et al., 2010), and characteristics of the therapist/instructor (Baldwin and Imel, 2013). Since these common factors are all related to the social and relational aspects of therapy and are the focus of the present study, we will hereafter refer to them as social common factors.

Only a handful of MBI studies have explored the impact of therapeutic alliance on outcomes and they vary widely by outcome and type of intervention. Goldberg et al. (2013) investigated the relationship between therapeutic alliance and outcome variables in an MBI for smoking cessation. Measures of alliance were associated with improvements in self-reported mindfulness, psychological distress, and emotion regulation, with similar effect sizes as have been found in other treatments. Similarly, Bowen and Kurz (2012) found therapeutic alliance to predict increases in self-reported mindfulness in a study on MBIs for substance abuse. Day et al. (2016) investigated common and specific factors in a small sample of MBCT for headache reduction, and found that therapeutic alliance predicted greater patient satisfaction but not reductions in pain interference. Jazaieri et al. (2018) found that therapeutic alliance predicted social anxiety reduction in MBSR but not in a CBT comparison group. In MBCT for cancer patients, Bisseling E. et al. (2019), Bisseling E. M. et al. (2019) found that therapeutic alliance but not group coherence significantly predicted reductions in psychological distress and improvements in wellbeing for both group-based-MBCT and internet-based-MBCT. However, none of these studies have investigated the impact of common factors on multiple outcomes in a sample of individuals with depression or anxiety.

The importance of the group in MBIs has been studied less than therapeutic alliance. While Bisseling E. M. et al. (2019) found that group coherence did not significantly predict reductions in psychological distress, other studies suggest that group therapeutic factors may play an important role in MBIs. Qualitative studies on the experience of participants in MBIs have found evidence for a core theme of the “normalizing and supportive influence of the group” (Wyatt et al., 2014). Additionally, a qualitative study for participants with academic evaluation anxiety (Hjeltnes et al., 2015) revealed instillation of hope (Yalom, 2005) as an important theme that included the sharing of human struggling in a group. From a quantitative perspective, Imel et al. (2008) found that group level variance in a multilevel model explained 7% of the variance of an MBSR class on psychological distress. Thus, further quantitative and qualitative research on the importance of an array of group therapeutic factors for the outcomes may yield important novel findings.

As part of a larger project that dismantled MBCT into single component treatments consisting of different meditation practices to investigate underlying mechanisms (see Britton et al., 2018), this paper aims to analyze and compare the influence of both common and specific therapeutic factors on changes in the outcomes of an MBI. Instructor and group related social therapeutic factors were used as examples of common therapeutic factors, while formal and informal mindfulness meditation practice were used as examples of specific therapeutic factors. We also investigated whether the presence of different instructor-specific treatments and therapy groups nested within them might be associated with participant’s assessments of social factors and/or moderate the effects of meditation minutes on changes in outcomes. Dependent variables consisted of changes in depression, anxiety, stress, and self-reported mindfulness. A mixed-methods quantitative and qualitative design was used to add rich participant description of social common factors to statistical inference.

## Materials and Methods

### Participants

English-speaking individuals between the ages of 18 and 65 with mild to severe levels of depression and anxiety were recruited from the Providence, RI, United States area. Exclusion criteria were: lifetime history of bipolar, psychotic, borderline or antisocial personality disorders, repeated self-harm or organic brain damage; current depression in the extremely severe range or active suicidal ideation; current panic, post-traumatic stress disorder, obsessive-compulsive disorder, eating disorder or substance abuse; current psychotherapy; a regular meditation practice or addition or modification of antidepressant medication in the last 2 months. See Britton et al. (2018) for details.

### Setting and Oversight

The registered clinical trial (clinicaltrials.gov #NCT01831362) took place at Brown University between November 2012 and March 2016, and was approved and supervised by the Brown University Institutional Review Board, an independent Data Safety Monitoring Board and the National Center for Complementary and Integrative Health’s Office of Clinical and Regulatory Affairs. Participants were recruited through community flyers advertising meditation for stress, anxiety and depression. Eligible participants provided written, informed consent approved and did not receive financial compensation.

### Interventions

As part of the primary dismantling study (Britton et al., 2018), participants were randomized to either focused attention (FA), open monitoring (OM), or standard MBCT. The MBCT module followed the specification of the published session-by-session manual with standardized handouts (Segal et al., 2002), while the FA and OM curriculums were single component variants of MBCT that emphasized a specific type of meditation. MBCT includes aspects of both FA and OM and was the primary treatment dismantled in this trial (see Britton et al., 2018 for more details about the contents of each intervention). For each intervention, 3-h classes were held once per week for 8 weeks, including a full silent retreat day in either week 6 or 7. All three intervention groups were closely matched in overall structure and duration such that they only importantly differed in terms of the types of meditation techniques that were taught.

### Instructors

All groups were instructed by a female and a male instructor. While all instructors had 20 years of meditation practice, age and clinical experience differed. The female trained MBSR/MBCT instructor and clinical psychologist (Instructor 1, age 38) taught in all groups and was accompanied by a different male instructor in each treatment condition, who specialized in the specific intervention. Instructor 2 (age 33, OM condition) was a former ordained Theravādan Buddhist monk and meditation teacher who specialized in the noting-style of vipassana and had no clinical training. Instructor 3 (age 52, FA condition) was the only full-time clinician, MBSR- and Dialectical Behavior Therapy-trained with more than 20 years of experience as a mental health counselor and a background of concentration practices of Theravāda Buddhism. Instructor 4 (age 39, MBCT condition) was a qualified MBSR instructor and research psychologist with Zen Buddhist meditation practice and teaching experience. See Britton et al. (2018) for more information on the background of the instructors.

### Quantitative Measures

#### The Empathy Scale (ES)

The Empathy Scale (α = 0.89; Persons and Burns, 1985; Burns and Auerbach, 1996) is a 10-item self-report questionnaire that assesses participants’ perceptions of the instructor’s warmth, genuineness, and empathy. Each item is scored on a 7-point Likert scale, with responses ranging from strongly disagree (0) to strongly agree (6).

#### The Working Alliance Inventory (WAI)

The Working Alliance Inventory (Horvath and Greenberg, 1989) measures therapeutic alliance in terms of tasks (therapeutic activities perceived as relevant and efficacious), goals (agreement on goals of treatment), and bond (mutual trust and rapport). This study, following the example of Johnson et al. (2005), used the 20-item version of the WAI, which was administered twice: once in reference to the group instructors (the Participant-Instructor WAI) and once in reference to the other group participants (the Participant-Participant WAI). The scale has three subscales: agreement on tasks (Participant-Instructor α = 0.87), agreement on goals (Participant-Instructor α = 0.78), and bond (Participant-Instructor α = 0.91; Participant-Participant α = 0.92). Only the bond subscale was used in reference to group members. Each item was rated on a 7-point Likert scale (not at all true, 1, to very true, 7).

#### The Therapeutic Factors Inventory-19 (TFI-19)

Group therapeutic factors were measured using the Therapeutic Factors Inventory-19 (TFI-19; Macnair-Semands et al., 2010; Joyce et al., 2011), a measure designed to assess member’s perceptions of the presence of four higher-order group therapeutic factors in group therapy: instillation of hope (hope for improvement as a result of the group; α = 0.90), secure emotional expression (safety in self-disclosure and emotional expression in group; α = 0.82), awareness of relational impact (insight into group interpersonal interactions and personal thoughts, feelings, and behaviors; α = 0.83), and social learning (skills learning through the group; α = 0.58). Items on the TFI are rated using a seven-point Likert scale ranging from strongly disagree (0) to strongly agree (6).

#### Formal and Informal Mindfulness Practice

Meditation homework was assigned as 45 min per day of formal meditation practice 6 days a week in all treatments. In addition, participants were assigned informal mindfulness exercises throughout the day, such as mindfully brushing teeth or walking. Throughout the intervention, the number of minutes practiced and frequency of informal practice was monitored through daily logs that participants filled out online through Survey Monkey. At 3-month-follow-up, participants filled out the amount and type of formal and informal mindfulness practice that they had practiced since post-course. Formal meditation practice minutes and informal mindfulness frequency were summed cumulatively for each week of the intervention and at follow-up.

#### Depression Anxiety Stress Scales (DASS)

The Depression Anxiety Stress Scales (Lovibond and Lovibond, 1995; Brown et al., 1997; Crawford and Henry, 2003) is a 42-item self-report questionnaire that measures depression (α = 0.92–0.95), anxiety (α = 0.77–0.85) and stress (α = 0.88–0.92). Each item is scored on a four-point scale (0 = *Did not apply to me at all*, to 3 = *Applied to me very much, or most of the time*).

#### The Five Facet Mindfulness Questionnaire (FFMQ)

The Five Facet Mindfulness Questionnaire (α = 0.91–0.94; Baer et al., 2006) is a 39-item mindfulness scale developed from a factor analysis of items from several preexisting mindfulness scales. The scale delineates mindfulness into five facets: observing, describing, acting with awareness, non-judging of inner experience, and non-reactivity to inner experience, which are summed together to form a total score, which we used in this study. It uses a 5-point Likert scale, with responses ranging from *never or very rarely true* (1) to *very often or always true* (5).

### Procedure

Participants filled out all survey measures online through Survey Monkey throughout the intervention and at 3-month-follow-up. The measures of social common factors (the ES, WAI, and TFI) were administered at post-intervention (week 8). The DASS was administered at baseline and every 2 weeks throughout the intervention (weeks 0, 2, 4, 6, 8), and 3-month-follow-up (week 20). The FFMQ was administered throughout the intervention (weeks 0, 3, 5, 7, 8) and at 3-month-follow-up (week 20). Formal and informal mindfulness practice was logged daily throughout the intervention (weeks 1–8) and again at 3-month-follow-up (week 20).

Qualitative interview data were gathered at 3-month-follow-up. Study participants were given structured interviews that queried continued meditation practice and impact of the treatment. Interviews were conducted by study personnel rather than treatment providers to minimize demand characteristics. Interviews were audio recorded and transcribed to facilitate qualitative content analysis using NVivo software. The complete interview protocol can be found in the supplementary material of Britton et al. (2018).

### Quantitative Analysis

All analyses were conducted using SPSS 25 and R 4.0. Only participants who completed the intervention were included in analyses. Multilevel growth curve models were constructed using the nlme R package with maximum likelihood estimation. This approach estimates slope and intercept parameters that model each individual’s trajectory of change and allow for nested data designs. Separate growth curve models were constructed to model longitudinal changes in depression, anxiety, stress, and mindfulness. Nesting was structured such that time (level one) was nested within each participant (level two), each participant was nested within one of the nine groups (level three), and each group was nested within the three treatment types (level four). Univariate and multivariate model assumptions were investigated for each model.

Growth models without predictors were constructed using an exploratory approach to find the best fitting and most parsimonious model for the effect of time on dependent variables. Model construction began with an unconditional mean model for each outcome to determine the intra-class correlation coefficient (ICC) for each level of the data. Random effects that accounted for no variance were dropped from models. Subsequently, linear and polynomial effects of time were fit to the data as fixed and random effects using deviance statistics to determine whether each additional model parameter improved the fit of the model. Only parameters that significantly improved the fit of the model were maintained in the growth curve model. Additionally, error and variance structures were fit to the data using deviance statistics to determine the most appropriate model.

Predictors were added to the growth curve models to predict change in the dependent variables. Dimension reduction through exploratory factor analysis using principal components extraction was used to reduce the number of related instructor and group variables. One-way ANOVA tests were used to test for differences in predictor variables between the three treatment types/instructors (MBCT, FA, and OM) and the nine treatment groups. Social common factors were added to the growth curve models as time-invariant fixed predictors of model slope and intercept and meditation minutes were added as time-variant predictors of change (see Singer and Willett, 2003). The time variable was measured in weeks, with post-course (8 weeks) set to 0 so that the intercept would be equivalent to the post-course mean.

### Qualitative Analysis of Social Common Factors

The qualitative analysis was derived from six questions addressing the impact and importance of the intervention to the participant’s life, how the participant changed because of the intervention, and the most valuable aspect(s) of the intervention. The importance of social factors in the intervention was directly queried in the fourth question. Participant responses that were unrelated to social factors were not included in this analysis.

Qualitative analysis of interview transcripts employed both a theory-driven and data-driven approach (DeCuir-Gunby et al., 2011) to creating codebook categories. The relationship to group categories were both data-driven, such that they emerged from the transcript content, and were inspired by Yalom’s (2005) framework for group therapy. The descriptions, inclusion criteria, and exclusion criteria of codebook categories were revised iteratively during the initial stages of the coding process until saturation was reached and no further data-driven categories emerged (Guest et al., 2012). Before study personnel applied the coding structure across all transcripts, two rounds of preliminary coding on a subset of transcripts allowed for discrepancies in coding to be discussed until consensus was reached. Codebook categories were applied to all references by two coders, and all discrepancies were discussed until consensus was reached.

## Results

### Demographics

One hundred and four participants were randomized into nine groups, consisting of 10–13 individuals each, three for each treatment type. A total of 96 participants completed the interventions and were included in analysis. Sample characteristics were matched across treatment type on age, gender, race/ethnicity, and baseline levels of psychopathology (see Britton et al., 2018). The total sample had a mean age of 40.3 ± 12.8, was 73% female, 99% White and 1% Asian, and 7% Hispanic/Latinx and 93% not Hispanic/Latinx. Highest levels of education for the total sample were 2.9% high school only, 53.8% college, 27.9% Master’s degree, and 15.4% doctoral degree. Criteria for current Major Depressive Disorder (MDD) was met by 39% of the sample, and 50% met criteria for Generalized Anxiety Disorder (GAD). The majority of the sample had either clinical or subclinical levels of GAD or MDD at the time of enrollment (85.6%), or in the past (93.3%). A third (33.7%) of the sample were taking antidepressant medication. Eighty-eight out of the final 96 participants had complete audio-recordings of the 3-month-follow-up interview.

### Quantitative Results

#### Preliminary Analyses

See Table 1 for the means and standard deviations of meditation minutes, informal mindfulness frequency, and outcome variables at each time measurement. See Table 2 for the means and standard deviations of instructor and group factors. Independent variables had no missing data and dependent variables (DASS and FFMQ) had 2.60–3.30% missing data when considering all six time points. All participants had a minimum of three time points present in the data. Missing data was handled with the maximum likelihood procedure in the mixed effects models. No extreme outliers were identified.

**TABLE 1:** Descriptive statistics for time-variant predictor and outcome variables.

	Time in weeks										
Variables		0	1	2	3	4	5	6	7	8	20
Depression	*M*	23.53	–	21.74	–	20.28	–	19.39	–	18.35	19.30
	*SD*	7.72	–	7.75	–	6.89	–	5.46	–	5.38	6.98
Anxiety	*M*	18.66	–	18.95	–	18.04	–	17.99	–	16.92	16.88
	*SD*	4.21	–	3.87	–	4.05	–	4.09	–	3.19	3.96
Stress	*M*	28.32	–	26.75	–	24.90	–	23.77	–	22.84	22.59
	*SD*	7.04	–	7.04	–	6.72	–	6.05	–	6.10	6.78
Mindfulness	*M*	122.77	–	–	123.08	–	129.67	–	138.37	144.06	144.86
	*SD*	18.93	–	–	16.75	–	18.12	–	18.88	18.56	19.92
Cumulative meditation minutes	*M*	0.00	231.72	448.16	671.60	883.19	1093.28	1298.17	1461.30	1620.37	2826.89
	*SD*	0.00	78.31	147.75	224.40	299.67	377.61	466.74	528.68	589.77	1537.17
Cumulative informal mindfulness frequency	*M*	0.00	3.57	6.92	12.23	18.07	21.93	25.55	28.41	30.73	88.36
	*SD*	0.00	4.23	7.61	10.30	13.69	15.60	18.11	20.14	22.11	78.77

*Week 0 represents baseline, week 8 represents post-course, and week 20 represents 3-month-follow-up. Depression, anxiety, and stress were measured with the Depression, Anxiety, and Stress Scales (DASS). Mindfulness was measured with the Five Facet Mindfulness Questionnaire (FFMQ).*

**TABLE 2:** Descriptive statistics for instructor and group related time-invariant predictors.

	*M*	*SD*
**Instructor:**		
Empathy Scale	62.72	8.51
WAI leaders bond	69.43	10.45
WAI leaders task	21.58	4.72
WAI leaders goal	22.09	4.30
**Group:**		
WAI members bond	65.61	10.76
TFI hope	5.58	1.13
TFI expression	5.21	0.96
TFI relational	4.30	1.32
TFI social	4.32	1.18

*WAI, working alliance inventory; TFI, therapeutic factors inventory. Scales were measured at post-course (week 8).*

Correlation analyses revealed that the four instructor factors (ES, WAI leaders bond, task, and goal) had a range of bivariate correlations between *r* = 0.47 and *r* = 0.78, while the five group factors (WAI members bond, TFI hope, expression, relational, and social) had a range of bivariate correlations between *r* = 0.50 and 0.78. Exploratory factor analysis using principal components extraction was used to reduce the number of teacher and group related factors. Only factors with an eigenvalue above 1.0 were retained. For teacher variables, one factor emerged, which explained 73.16% of the variance of the four measures. For group variables, one factor emerged, which explained 68.19% of the variance of the five measures. These factors were saved in the dataset using the Bartlett approach.

The instructor ratings factor significantly differed by treatment type, *F*(2,93) = 7.18, *p* = 0.001, but did not significantly differ by group, *F*(8,87) = 2.04, *p* = 0.051. Bonferroni-adjusted *post hoc* tests found that the FA treatment type/instructor had the highest mean instructor ratings, while the OM treatment type/instructor had the lowest mean instructor ratings. FA and OM mean instructor ratings were significantly different, *M*_*d*_ = 0.87, *SE* = 0.23, *p* = 0.001, while neither was significantly different from the MBCT treatment type/instructor. Group ratings did not significantly differ by treatment type, *F*(2,93) = 2.40, *p* = 0.096, or by group, *F*(8,87) = 1.73, *p* = 0.102. Total formal meditation minutes from baseline to follow-up did not differ by treatment type, *F*(2,93) = 0.30, *p* = 0.743, or by group, *F*(8,87) = 0.96, *p* = 0.475. Total frequency of informal mindfulness practice from baseline to follow-up did not differ by treatment type, *F*(2,93) = 0.24, *p* = 0.789, or by group, *F*(8,87) = 1.20, *p* = 0.311.

#### Construction of Growth Curve Models

Examination of model residuals revealed non-normal residuals and heteroscedasticity in models predicting depression and anxiety. Depression scores at all time points (skew: 1.65, kurtosis: 3.04) and anxiety scores at all time points (skew: 1.67, kurtosis: 3.68) were not normally distributed. As a result, Box-Cox power transformations were used to find an optimal transformation to a normal distribution for these variables as based on maximum likelihood estimation (Pek et al., 2018). The Box-Cox power transformation estimates were −1.65 for depression and −2.38 for anxiety, both of which were rounded to −2 to ease interpretability. Since the transformations of x^–2^ yielded very small values, a constant was added to the transformations so that the transformed values would have a similar range as the untransformed values for depression and anxiety. This led to a transformation for depression of 8000/x^2^ (range 2.55–40.82, *M* = 24.33, *SD* = 11.25) and a transformation for anxiety of 6000/x^2^ (range 3.75–30.61, *M* = 20.91, *SD* = 6.95). Note that for the transformed variables, higher scores indicate lower depression and anxiety.

Table 3 reports the parameters of the growth curve models that were constructed for depression, anxiety, stress, and mindfulness scores before predictors were added to the models. These models describe the effects of time and the nested structure of the data on each of the four outcomes. Note that a lack of group or treatment variance indicates that there was no effect of the nine groups or three treatment types on the outcome (depression, anxiety, stress, or mindfulness). Furthermore, significant linear, quadratic, and/or cubic time coefficients indicate that the outcome (depression, anxiety, stress, or mindfulness) significantly changed throughout the intervention and follow-up period.

**TABLE 3:** Growth curve models without predictors of change.

	Model 1: Depression	Model 2: Anxiety	Model 3: Stress	Model 4: Mindfulness
Variable	*B (SE)*	*B (SE)*	*B (SE)*	*B (SE)*
Intercept	27.29*** (1.16)	22.57*** (1.17)	22.52*** (0.57)	143.61*** (1.92)
Time	0.65*** (0.11)	0.74*** (0.13)	−0.42*** (0.04)	4.81*** (0.41)
Time^2^	−0.06*** (0.01)		0.03*** (0.01)	
Time^3^		−0.005*** (0.001)		−0.03*** (0.003)
Variances				
σ*^2^* residual	3.03	31.47	0.01	78.98
σ*^2^* participant intercept	62.77	18.31	20.70	298.64
σ*^2^* participant time	0.05			9.22
*R* (participant σ*^2^* intercept, participant σ*^2^* time)	0.40			0.51
σ*^2^* participant time^3^				0.0005
*R* (participant σ*^2^* intercept, participant σ*^2^* time^3^)				−0.47
*R* (participant σ*^2^* time, participant σ*^2^* time^3^)				−0.99
σ*^2^* group intercept	3.40			
σ*^2^* group time	0.07			
*R* (group σ*^2^* intercept, group σ*^2^* time)	−0.46			
σ*^2^* treatment intercept		3.04		
Deviance	3923.55	3477.95	3422.15	4406.33
AIC	3947.55	3501.95	3436.15	4426.33

***p < 0.001. Higher scores on anxiety and depression scales indicate less anxiety and depression due to box-cox transformation. The time variable was coded such that post-course scores represent the model intercept.

The construction of these models began by investigating the nested structure of the data. Unconditional mean models for each outcome with random intercepts for participant, group, and treatment were constructed. Random effects that did not explain any variance were removed (ICC < 0.001). For depression scores, 49% of variance was due to individual differences, 3% was due to group differences, and 0% was due to treatment differences. For anxiety scores, 42% was due to individual differences, 0% was due to group differences, and 7% was due to treatment differences. For stress scores, 47% of the variance was due to individual differences, 0% was due to group differences, and 0% was due to treatment differences. For mindfulness scores, 50% of the variance was due to individual differences, 0% was due to group differences, and 0% was due to treatment differences. Interestingly, this means that variation between treatments, when collapsing across all time points, was only found for anxiety scores.

Following this, tests for linear and polynomial effects of time were added to each model and retained when they significantly improved model fit. The growth curve models that provided the best fit to the data had linear and quadratic time coefficients (fixed effects) for depression and stress and linear and cubic time coefficients (fixed effects) for anxiety and mindfulness. Quartic time coefficients were tested but were not significant for any of the models. These models describe the average curvilinear relationship between time and each outcome as a quadratic or cubic equation. Note that time is measured in weeks and centered at post-course. According to the models (see Table 3), average depression scores declined from pre-course to post-course, and stayed approximately the same from post-course to follow-up. Average anxiety scores were flat for the first 2 weeks and then declined at an accelerating rate to post-course, then stayed approximately the same from post-course to follow-up. Average stress scores had a gradually accelerating rate of decrease from pre-course to post-course to 3-month-follow-up. Average mindfulness scores had an accelerating rate of increase from pre-course to post-course, followed by a slight increase from post-course to 3-month-follow-up. All four outcomes significantly changed.

Random slopes were also tested for linear and polynomial effects of time at each level of the nested data. See Table 3 for variance estimates and correlations between variance estimates. Allowing variation in the linear slopes across individual participants (σ*^2^* participant time) only significantly improved the models for depression and mindfulness scores, while allowing linear slopes to vary across the nine groups (σ*^2^* group time) only significantly improved the model for depression scores. Allowing variation in quadratic slopes did not improve model fit at any level of analysis, while allowing variation in the cubic slopes at the individual participant level (σ*^2^* participant time^3^) significantly improved the fit of the model for mindfulness scores. The models for anxiety and stress scores were not significantly improved by including any random slopes. Furthermore, none of the models were significantly improved by including variation in treatment slopes. Interestingly, this means that treatment type had no effect on changes in depression, anxiety, stress, or mindfulness scores.

Finally, error and residual variance structures were fit to each model. An autocorrelated error structure significantly improved model fit for the depression (*p* = 0.006, phi = 0.20), anxiety (*p* < 0.001, phi = 0.22), and stress (*p* < 0.001, phi = 0.20) score models. A variance function consisting of the power of the fitted values was the best fit to the data for the depression (*p* < 0.001, power = 0.43), and stress (*p* < 0.001, power = 1.18) score models, while the best fitting variance function for the anxiety score model estimated variances at each time point. Tests for error and residual variance structure did not converge or did not significantly improve model fit for the mindfulness score model.

#### Assessing Predictors of Change

Table 4 reports the results of adding predictors of change to the growth models. The individual and group factors were added as time-invariant predictors of post-course intercept and model slopes (linear and quadratic or linear and cubic). Cumulative meditation minutes and informal mindfulness frequency were added to the models as time-variant predictors.

**TABLE 4:** Predictors of changes in depression, anxiety, stress, and mindfulness.

		Depression			Anxiety			Stress			Mindfulness		
Predictors:		Deviance (df)	*b*	*SE*	Deviance (df)	*b*	*SE*	Deviance (df)	*b*	*SE*	Deviance (df)	*b*	*SE*
Instructor factor		19.30*** (3)			4.78 (3)			22.22*** (3)			6.98∼ (3)		
	Post-course intercept		4.02***	0.92		1.35*	0.60		−2.32***	0.56		4.38*	1.89
	X time		0.93**	0.36		0.62	0.77		−0.61*	0.26		3.26	2.43
	X time^2^		−0.54	0.37					0.60*	0.28			
	X time^3^					−0.53	0.65					−2.05	1.97
Group factor		7.37∼ (3)			3.94 (3)			15.78** (3)			17.27*** (3)		
	Post-course intercept		2.26*	0.97		0.98	0.60		−1.73**	0.56		7.32***	1.78
	X time		0.66∼	0.37		0.83	0.78		−0.65**	0.25		5.85*	2.38
	X time^2^		<−0.22	0.39					0.39	0.27			
	X time^3^					−0.51	0.65					−4.10*	1.94
Formal Meditation Minutes ÷ 1,000		0.32 (1)	0.49	0.58	4.12* (1)	0.71*	0.35	7.38** (1)	−0.71**	0.27	2.29 (1)	1.26	0.82
Informal Mindfulness Frequency ÷ 100		1.11 (1)	−0.85	1.00	<0.01 (1)	0.03	0.67	0.14 (1)	−0.12	0.55	1.77 (1)	−2.14	1.53

∼*p* < 0.10, *p < 0.05, **p < 0.01, ***p < 0.001. Higher scores on anxiety and depression scales indicate less anxiety and depression due to box-cox transformation. For time-invariant predictors, interactions with effects of time refer to predictions of change, while time-variant predictors were entered directly into the model. Time variables were divided by their standard deviation so that interactions with each exponent of time would be on the same scale. All time variables were coded such that the model intercept is the post-course time-point.

##### Instructor ratings

The addition of the instructor factor as a predictor of post-course intercept and linear and quadratic slope into the growth models for depression and stress was significant. For the depression model, greater instructor ratings were associated with less depression at post-course and a steeper linear decline in depression, but were not associated with the level of curvature in the growth curve line as represented by the quadratic slope. Pseudo *R*^2^ statistics indicated that when compared to the growth curve model for depression without predictors (see Table 3), the instructor factor explained 15% of the participant intercept variation (σ*^2^* = 53.50), 20% of the participant slope variation (σ*^2^* = 0.04), and 95% of the group intercept variation (σ*^2^* = 0.16). These Pseudo *R*^2^ statistics should be interpreted with caution as group linear slope variance increased by 14% (σ*^2^* = 0.08) and residual variance increased by 3% (σ*^2^* = 3.13).

For the stress model, greater instructor ratings were associated with less stress at post-course, steeper rates of stress reduction as determined by the linear slope, and greater levels of curvature in the line as measured by the quadratic slope. Pseudo *R*^2^ statistics indicated that when compared to the growth curve model for stress without predictors (see Table 3), the instructor factor explained 15% of the participant intercept variation (σ*^2^* = 17.65), and 49% of the residual variation in the model (σ*^2^* = 0.01).

For the anxiety and mindfulness models, greater instructor ratings were associated with less anxiety and greater mindfulness at post-course, but were not associated with linear or cubic slopes.

##### Group ratings

The addition of the group factor as a predictor of post-course intercept and slopes (linear and quadratic or linear and cubic) into the growth models for stress and mindfulness was significant. For the stress model, greater group ratings significantly predicted lower post-course stress ratings, and greater reductions in stress as measured by the linear slope. Group ratings were not significantly related to the curvature in the line, as measured by the quadratic slope. Pseudo *R*^2^ statistics indicated that when compared to the growth curve model for stress without predictors (see Table 3), the group factor explained 9% of the participant intercept variation (σ*^2^* = 18.76), while residual variation in the model increased by 4% (σ*^2^* = 0.01).

For the mindfulness model, greater group ratings significantly predicted greater post-course mindfulness ratings, greater increases in mindfulness as measured by the linear slope, and greater levels of curvature in the line as measured by the cubic slope. Pseudo *R*^2^ statistics indicated that when compared to the growth curve model for mindfulness without predictors (see Table 3), the group factor explained 18% of the participant intercept variation (σ*^2^* = 245.52), 11% of linear slope variation (σ*^2^* = 8.23), 9% of cubic slope variation (σ*^2^* = < 0.01) and 0% of residual variation in the model (σ*^2^* = 78.98).

For the depression model, greater group ratings were associated with lower depression scores at post-intervention, but were not associated with linear or quadratic slope. The group factor was not associated with the post-course intercept or slopes for anxiety.

##### Formal meditation practice

The addition of cumulative formal meditation practice minutes as a time-variant predictor into the models for anxiety and stress was significant, but was not significant for the depression and mindfulness models. For anxiety, the cumulative amount that participants meditated was associated with lower anxiety when considering all time points (*b* = 0.001, *SE* = 0.0003, *p* = 0.043). Pseudo *R*^2^ statistics indicated that when compared to the growth curve model for anxiety without predictors (see Table 3), cumulative meditation minutes explained < 0.01% of the participant intercept variation (σ*^2^* = 18.29), and 1% of the residual variation in the model (σ*^2^* = 31.11), whereas the variance in treatment increased by 5% (σ*^2^* = 3.19). For stress, the cumulative amount that participants meditated was associated with lower stress across all time points (*b* = −0.001, *SE* = 0.0003, *p* = 0.009). Pseudo *R*^2^ statistics indicated that when compared to the growth curve model for stress without predictors (see Table 3), cumulative meditation minutes explained 4% of the participant intercept variation (σ*^2^* = 19.84), and 38% of the residual variation in the model (σ*^2^* = 0.01).

Since the three treatment types practiced different forms of meditation, treatment type was also added to each model as a dummy coded interaction term with meditation minutes. This did not improve model fit for any of the models (depression: χ*^2^_*Change*_* (4) = 3.14, *p* = 0.535; anxiety: χ*^2^_*Change*_* (1) = 3.37, *p* = 0.066; stress: χ*^2^_*Change*_* (4) = 4.37, *p* = 0.358; mindfulness: χ*^2^_*Change*_* (4) = 5.20, *p* = 0.267), and none of the dummy coded treatment-meditation interaction terms were significant.

##### Informal mindfulness frequency

The addition of cumulative informal mindfulness practice frequency as a time-variant predictor was non-significant for all models. Within these models, informal mindfulness practice frequency had no relationship to changes in depression, anxiety, stress, or mindfulness.

#### Predictor Comparisons

Akaike information criteria (AIC) statistics were used to compare models with different predictors such that lower AIC values indicate better model fit. For depression, the instructor ratings factor provided the best fit to the data (AIC = 3934.25) followed by the group ratings factor (AIC = 3946.18). Both instructor and group had better fit than the depression growth model without predictors (AIC = 3947.55), while informal mindfulness frequency (AIC = 3948.46) and formal meditation minutes (AIC = 3949.23) had worse fit.

For anxiety, the model with formal meditation minutes provided the best fit to the data (AIC = 3499.84), which was the only predictor leading to a better fit than the growth model without predictors (AIC = 3501.95). The instructor factor (AIC = 3503.17), informal mindfulness frequency (AIC = 3503.95), and the group factor (AIC = 3504.01) resulted in similarly poor model fit.

For stress, the model with the instructor ratings factor provided the best fit to the data (AIC = 3419.93), followed by the model with the group ratings factor (AIC = 3426.38), and the model with formal meditation minutes (AIC = 3430.77). Only the model with informal mindfulness frequency (AIC = 3438.01) had a worse fit than the stress growth model without predictors (AIC = 3436.15).

For mindfulness, the model with the group ratings factor provided the best fit to the data (AIC = 4415.06), followed by the model with the instructor ratings factor (AIC = 4425.35). The models with formal meditation practice (AIC = 4426.04) and informal mindfulness frequency (AIC = 4426.56), had approximately the same fit as the mindfulness growth model without predictors (AIC = 4426.33).

### Qualitative Results

**FIGURE 1:**
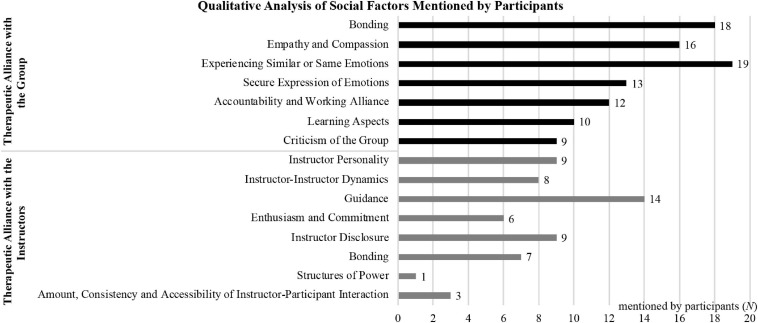
Frequency of teacher and group related social factors mentioned by participants.

Eighty-eight participants provided qualitative interviews. Among these, 67 (76%) mentioned group social factors and 32 (36%) mentioned instructor social factors as active ingredients of the program (see Figure 1). Social factors reported during these interviews include five types of therapeutic alliance with the instructors and six types of therapeutic alliance with the group. In addition, participants also provided a critical perspective on the influence of instructors and the group.

#### Relationship to Group

##### Bonding

Eighteen participants (20%) stressed the importance for them of bonding with the group in terms of generating a sense of community, closeness, (emotional) connection and inclusion; a feeling of being cared for; and a protective environment for openly sharing with and being compassionate to each other. One participant noted that “the sense of community gives people belonging and gives people more comfort in accepting and trying something new.” Participants also made connections between bonding and recognizing that they are not alone in their struggles. For example, one participant stated that “I always feel less alone, more connected, realizing that there are other people struggling with the same issues that I have.” Other participants reported making friends in the class or feeling “a depth of connection with them” when encountering them outside of class.

##### Empathy and compassion

Sixteen participants (18%) observed that everybody was affected differently, that “everybody has their own things,” and that other people also struggled. These observations about the group increased participants’ compassion, and helped them to be “more kind and considerate to others.” One participant noted that “the interaction between the people within the group was extremely helpful because you learn from other people’s experiences,” hearing “what they were doing and how they’d used it in their personal life […] was very meaningful.” Others stressed how the group was courageous in their honesty and “open about their vulnerabilities.”

##### Experiencing similar or same emotions

Nineteen participants (22%) mentioned experiencing similar or same emotions, which included struggling with the same issues. Participants also acknowledged how talking about these struggles with each other every week helped them to keep from giving up “because it’s nice to feel like you’re not going through this alone.” Furthermore, “most people need to be part of the group” because “it’s very, very difficult to do it on their own.” Numerous participants used the phrase “going through” to signal the perception of a shared experiential process or similar emotions. Validation of one’s own “experiences, fears, limitations,” feelings, emotions or universal problems, feeling that participants were “in the same boat,” and “normalizing mental distress” were also cited as important aspects of the course.

##### Secure expression of emotions

Being able to securely express emotions was appreciated by thirteen participants (15%), because “that helped us all relate to each other and connect and get through this process together.” “Everybody felt really comfortable” and in “a very nice environment.” Participants were “willing to talk about deeply personal issues.” This openness was possible because of “trust,” a “palpable respect among the participants,” a non-judgmental “atmosphere” of acceptance. Recognizing a degree of feeling “comfortable with each other” was essential to openness within the group, being open and vulnerable was also sometimes challenging.

##### Accountability and working alliance

Twelve participants (14%) mentioned various ways in which they were held accountable, including the homework and doing the practices. However, most of the comments concerned accountability with respect to the group. Participants felt that the structure of the group and the routine of meeting weekly were helpful, as they “owed it to them to be present.” Some participants stated that they needed the group for “remaining active,” to put time aside, or to solidify the meaningfulness of the practice.

##### Learning aspects

Ten participants (11%) appreciated learning about others’ experiences, especially about “how they’d used [mindfulness] in their personal life.” Given that each program included multiple practices, participants were often interested to hear the impacts and benefits associated with specific practices. Such statements were encouraging for some participants, and others framed them as adding “to my experience or my understanding or my appreciation” of the practice.

##### Criticisms of the group

However, nine participants (10%) also reported negative or critical aspects of the group. Two of these participants expressed difficulties connecting with the group on account of a significant difference in age from the other participants, which for one “was a bit of a barrier to maybe making friends.” Other participants said the group aspect “felt a little forced” or compared the challenge of “having to talk to people about my experience” to “being in high school again. I just felt like I was sort of on the sidelines.” One participant was critical that there was too much discussion during group time, or that certain participants “dominated the class” and were “the center of attention,” with the result that this participant put her head like a turtle back into her “own shell.”

#### Relationship to Instructor or Therapeutic Alliance With Instructor

##### Instructor personality

Nine participants (10%) described the instructors’ personalities as influential upon their experience. For example, they identified the temperament of Instructor 1 as “hilarious,” “down to earth,” and “realistic.” Hence, Instructor 1 was also perceived as confident and self-accepting which helped three participants in their progress. One participant noted about Instructor 1 that “when she admits her faults, you see she’s human.” One participant referred to Instructor 2’s biography and described him being more “a translator, he wasn’t a facilitator in this kind of thing.” Instructor 3 was described as “the sweetest, kindest, nicest person I’ve ever met” and “so calm and relaxing.” Instructor 4 was not described individually in his personality.

##### Instructor-instructor dynamics

Eight participants (9%) also described how the two instructors in their group contrasted or complemented each other. Some mentioned the value of having two instructors because they brought different personalities, approaches and perspectives, such as Instructor 1 and Instructor 4. One participant criticized the dynamic between Instructor 1 and Instructor 2, who “had a tendency to [.] bicker, and I thought that that was really unprofessional.”

##### Guidance

Fourteen participants (16%) commented on the instructors’ general skill sets and their experiential approach toward teaching meditation, being non-judgmental and dealing with difficult emotions. Participants perceived positively the invitation to choose freely among practices, a sense of help in the form of care or compassion, and how the instructors communicated their knowledge and prepared course materials from a scientific perspective. Participants also stated that the instructors provided good descriptions and explanations, using quotes, concrete suggestions, neuroscientific background, and easy to understand examples.

##### Enthusiasm and commitment

The instructor’s enthusiasm and commitment to the practice and group were mentioned by six participants (7%). Instructors were described as “dedicated” and “engaged” and “enthusiastic.” One participant mentioned the amount of joy and playfulness the class had to offer.

##### Instructor disclosure

Instructors’ self-disclosing about their own experiences was another approach that helped facilitating a sense of similarity between instructor and nine participants (10%). For example, participants appreciated hearing one instructors disclose “‘I was struggling so much’, it’s like: ‘Oh good, it’s not just me.”’ Others described benefiting from knowing that “there is someone who feels the same” as they do.

##### Bonding

Bonding was apparent in seven participants (8%), indicated by the resemblance felt between instructor and participant, a feeling of love, or of closeness. Participants appreciated how being “around people that have similar issues […] makes me feel more hopeful,” or how an instructor “really made it very comfortable for people to say anything.” In contrast, another instructor was criticized by a participant about not being able to facilitate open conversations because the instructor did not share personal stories. One participant described a lack of bond, saying “I didn’t feel this caring warmth coming from them in a way that would have made me feel safer.”

##### Structures of power or constraint

Only one participant (1%) commented on the power dynamic between the instructors and the participants, explaining it by contrasting participants’ vulnerability and the structure the instructors provided. This makes the instructor responsible “because you’re with a bunch of strangers and the only really solid structure is the instructor.”

##### Amount, consistency or accessibility of instructor-participant interaction

Instructor availability and accessibility was also rarely mentioned as an active ingredient. While two participants found it helpful to be able to ask questions during the course, another participant criticized the instructors’ lack of response and availability outside of the weekly meetings.

## Discussion

This project used a mixed-method design to investigate the contribution of social common factors to clinical outcomes of a Mindfulness-Based Intervention and compare them to the specific effects of mindfulness practice. The quantitative results from this study found that both instructor and group r elated social common factors and meditation minutes were important predictors of outcome changes in MBIs, while different styles of meditation had no effect on outcomes. The qualitative results expand on and illustrate the social factor findings, providing rich descriptions of the ways in which instructor and group social dynamics impacted participants.

### Treatment and Instructor Differences

This investigation took place in the context of a dismantling study of MBCT involving three treatment types that involved different forms of meditation practice and were taught by different instructors. Interestingly, while all four outcomes (depression, anxiety, stress, and mindfulness) significantly changed throughout the intervention and follow-up period, results indicated that the division into three treatment types did not account for any of the variance of changes in any of the outcomes. While treatment types accounted for some variance in anxiety scores when collapsing across all time points, no treatment type variation was found when considering the effects of time, indicating that the treatment type intercept variance in anxiety scores could be explained by baseline differences. Although the lack of differences in treatment types for outcome changes was not hypothesized, it is in line with the research questions of the present analysis given that forms of meditation practice are a form of specific effect. Thus, these findings suggest that the specific effects of forms of meditation, especially FA meditation in comparison to OM meditation, may not be important for intervention-related changes in depression, anxiety, stress, and mindfulness.

Treatment differences were found, however, for instructor ratings. The groups that were in the FA treatment condition with Instructors 1 and 3 showed significantly higher instructor ratings than the participants in the OM treatment condition with Instructors 1 and 2. As previously mentioned, Instructor 3 was the only full-time clinician, while Instructor 2 was a former Buddhist monk with no clinical experience or training. Since this study confounded treatment condition with instructors, differences in instructor ratings could stem from differences in the meditation practice or differences related to the instructors. However, given that these instructors differed especially on clinical experience, and participants in the groups with the instructor with the most clinical experience also had the highest instructor ratings, these results suggest that instructor’s clinical experience beyond mere meditation practice experience may be key to the development of social bonds and working alliances with students.

### Instructor Alliance

Within the quantitative findings, instructor ratings significantly predicted changes in depression and stress, but not anxiety or mindfulness. These findings are in line with the results of other studies on MBIs that found a relationship between therapeutic alliance with the instructor and psychological distress (Goldberg et al., 2013; Bisseling E. et al., 2019), yet our findings are more specific as we distinguish between depression, anxiety, and stress whereas other studies do not. For example, Goldberg et al. (2013), found a relationship between instructor alliance and the DASS total score, but did not examine the depression, anxiety, and stress subscales separately.

The qualitative data illustrated the importance of the relationship with the instructor for participants in their own words. Qualitatively, 8% of participants emphasized the importance of bonding with the instructor, specifically in the sense that instructors made them feel comfortable to self-disclose or participants felt cared for by instructors. Participants also emphasized the importance of instructor guidance (16%), enthusiasm and commitment (7%), and instructor self-disclosure (10%). Ten percent of participants mentioned the personality and experience of the instructor as important. Instructor humor, kindness, genuineness, confidence, self-disclosure, enthusiasm, commitment to the practice, engagement, and self-acceptance were all mentioned by participants as helpful characteristics.

Whereas Bowen and Kurz (2012) and Goldberg et al. (2013) found that instructor alliance predicted increased mindfulness in an MBI, the present study did not replicate their finding. Furthermore, our study did not find a link between therapeutic alliance and anxiety, indicating that other intervention components may be more important for anxiety reduction. A previous study on CBT therapy for individuals with social anxiety found no relation of working alliance with reductions in anxiety (Mörtberg, 2014). On the other hand, another study found a consistent link over time between therapeutic alliance and anxiety reduction in a group CBT (Norton and Kazantzis, 2016). Yet, when comparing group CBT with group MBSR for participants with social anxiety, working alliance was an important predictor of symptom reduction for MBSR not CBT (Jazaieri et al., 2018). As the above-mentioned results provide conflicting findings, further research is needed to understand the link between therapeutic alliance with the instructor in MBIs and specific symptom reduction.

### Group Therapeutic Factors

Our study also found that group ratings, including instillation of hope, bonding with members, secure emotional expression, awareness of relational impact and social learning significantly predicted changes in stress and mindfulness, but not in anxiety or depression. While measured with different outcomes than the findings of Bisseling E. et al. (2019), who found that group cohesion did not predict a reduction in psychological distress during an MBI, our results are in line with theirs considering that psychological distress is often synonymous with symptoms of depression and anxiety. We found a lack of an effect of group therapeutic factors on depression and anxiety, while group therapeutic factors specifically affected participant’s improvements in stress and mindfulness. While anxiety and depression are clinically relevant descriptions of several symptoms that might need specific help of a clinician, self-reported stress can be reduced especially when engaging with similarly stressed group members. Group dynamics and conversations on how to be mindful in daily life might lead to reporting greater mindfulness after treatment. Our study is the first to examine the impact of group therapeutic factors across different outcomes of an MBI, while differentiating between instructor and group, hence, more research is needed to understand the observed differences.

Qualitatively, many participants emphasized the importance of community, bonding and belonging when discussing the importance of the group. This is in line with previous qualitative findings emphasizing the importance of the group for support and validation (Wyatt et al., 2014). Realizing that the other participants were struggling as much and with the same problems and issues was reported as a powerful experience for many. Participants also mentioned a comfortable and non-judgmental atmosphere and a feeling of trust and respect between the group members that enabled participant’s willingness to talk openly, thus underscoring a link between bonding and secure emotional expression. Experiencing of similar emotions was described as important in that participant’s felt validated by understanding that they all experienced similar difficulties and could work through these difficulties together. Observing others’ struggles helped to normalize participant’s own issues. Furthermore, participants reported reductions in shame through the recognition that they were not alone in their suffering and that others experienced similar feelings and were able to develop self-acceptance of those feelings (such as in Hjeltnes et al., 2015).

Regarding empathy and compassion, participants gained insight into other participants’ problems and perspectives, which helped them to appreciate the practice of meditation more and to become more kind to each other. Some felt a willingness to help or a feeling of concern for each other. Thus, the group process in itself may support participant’s experiences of greater empathy and compassion for others, which has been found to increase through MBIs (Luberto et al., 2017). Finally, a few participants mentioned the value of learning about others’ experiences, especially regarding different meditation practices and their application to personal life.

### Mindfulness Practice

Formal meditation minutes were associated with changes in anxiety and stress, while unrelated to changes in depression and mindfulness. Informal mindfulness frequency was not predictive of any outcomes. The finding of a relationship between formal meditation practice and changes in anxiety and stress may be the result of the calming and grounding effects of meditative practices. Interestingly, however, the style of meditation did not moderate these relationships, as FA meditation is often considered to be a more calming and grounding practice than OM meditation, which is more insight oriented (Britton et al., 2018).

It is interesting that formal meditation minutes were unrelated to improvements in depressive symptoms, as MBCT is specifically designed to be effective for depression relapse reduction (Segal et al., 2002; Williams et al., 2014). Our findings suggest that the results of MBCT on depressive symptoms may instead be the result of common factors or the elements of cognitive therapy that are incorportated into MBCT. This would align with the findings of Williams et al. (2014), who found that MBCT with and without meditation were equally effective for depressive relapse prevention.

The findings that neither formal meditation minutes nor informal mindfulness practice was associated with changes in mindfulness scores, while changes in mindfulness were predicted by group ratings, is striking considering that self-reported mindfulness, by definition, is believed to be the result of mindfulness practice (e.g., Baer et al., 2008). Our findings, especially when combined with those of Visted et al. (2014), suggest that increases in self-reported mindfulness in MBIs may not specifically be the result of mindfulness practice and may often result from social common factors. This overall conclusion is supported by both Bowen and Kurz (2012) and Goldberg et al. (2013), who both found that therapeutic alliance predicted self-reported mindfulness improvements in an MBI. However, this conclusion is not entirely consistent with previous research, indicating that further investigation is needed to resolve disparate findings. For example, Bowen and Kurz (2012) found a relationship between home meditation minutes and mindfulness at post-treatment, while Baer et al. (2008) found that meditation experience in a sample of long-term meditators was correlated with four out of the five FFMQ subscales. Additionally, long term meditation practice could have a greater effect on self-reported mindfulness than short term practice. While further research is needed in this area, the presence of social elements in nearly all aspects of contemplative instruction may make separating social common factors from the effects of meditation a challenging endeavor.

Model comparisons revealed that social common factors exerted greater influence on changes in depression, stress, and mindfulness than specific mindfulness practice-related factors, while formal meditation exerted a greater influence on changes in anxiety than social common factors. These results suggest that MBIs may be effective due to both common and specific factors (i.e., social common factors and meditation), although for all outcomes except for anxiety, common factors exerted stronger effects. Our finding about the strength of social common factors challenges the typical assumption that meditation practice itself is the main active ingredient in MBIs such that more practice should be more beneficial for participants. This assumption is further challenged when our results are taken together with the previously mentioned comparisons of MBIs with similarly effective active control groups lacking meditation (MacCoon et al., 2012; Rosenkranz et al., 2013; Williams et al., 2014; Shallcross et al., 2015) and the findings of small to non-existent relationships between home meditation practice during MBIs and outcomes (Parsons et al., 2017).

A common factors contextual view of meditation practice could conceptualize it as one aspect of a therapeutic context that may facilitate therapeutic processes but may not be responsible for them in isolation. Assuming that every treatment component is independent - as the approaches of quantitative methods do - is in contrast to a holistic and contextual model of therapeutic effectiveness that emphasizes the therapeutic experience as a whole. Throughout these courses, meditation experiences are necessarily embedded in a social and psychoeducational context. According to a common factors perspective, meditation could be conceptualized as a form of the common factor that Laska et al. (2014) describe as “a set of procedures [.] that leads the patient to enact something that is positive, helpful, or adaptive” (p. 469).

### Implications

This study offers both clinical and research implications. The importance of instructor and group factors could have implications for the structure of MBIs, as well as the lack of importance of informal mindfulness practice and formal meditation for some outcomes. Williams et al. (2014) in particular found that MBCT without meditation (with 36 h less engagement) had equivalent outcomes. These results identify the need for additional research evaluating the optimal amount of meditation practice for MBIs. If MBIs are as effective with less formal meditation, whether at home or in class, programs could be streamlined to optimize accessibility, costs, and time. Mindfulness teacher training should include basic group organization skills to enhance the effect, e.g., by aiming to enhance adaptive processes within the group and fostering interaction between group members. Depending on the symptoms a participant joins an MBI with, they may need a different focus. Joining for merely stress reduction, the intervention could be streamlined and the focus could be on the group coherence. Participants that are anxious might benefit most from out-of-class meditation practice. For depressive symptoms, instructor alliance may be an important aspect, so clinical expertise could be crucial for participant outcomes. And if a participant is interested primarily in learning mindfulness skills, a regular group attendance might be more beneficial than home practice. Informal mindfulness practice may not be an important ingredient of MBIs for any of the outcomes measured in this study.

Furthermore, the present study indicates that the results of MBIs should not be generalized to meditation apps or other forms of meditation that occur in the absence of an instructor or group. While meditation apps largely assume that mindfulness practice or technique directly leads to mental health improvements, the present study suggests that this may only be true for improvements in anxiety, while interpersonal relationships may be key to other forms of mental health improvement. This could mean that meditation apps would benefit from building community or interactions between their users to increase their efficacy.

### Limitations

This study cannot conclude a causal relationship between the social common factors and the treatment outcomes because it did not experimentally change the amount or quality of social common factors. Even a reverse directionality is possible (Goldberg et al., 2013), such that a change in affect, stress and anxiety could lead to a more positive and important experience of the group, instructor and social support.

One potential criticism of the finding of a small effect for home meditation minutes could be insufficient variance in the variable. Participants were instructed to practice 45 min 6 days a week, leading to a total of 2160 min (36 h) recommended practice. Nevertheless, the range of the recorded practice was between 300 and 3160 min with a broad standard deviation of 590 min, which should be enough variance to explain outcomes. On the other hand, even though there was high variability, the lowest number still represents a good amount of practice. Our study suggests that it may not matter for outcomes how many minutes at home one formally or informally practices, however, if some participants had engaged in little to no practice, perhaps different results would have been seen. An experimental manipulation of the amount of participant home practice would be required to make a firm conclusion regarding this claim.

Furthermore, as Rosenkranz et al. (2019) explain, this claim may depend on what outcomes are measured, and specific process measures may be more sensitive to the effects of differential treatment components such as meditation. Our study used the FFMQ as an outcome, but further studies could use additional process measures that may detect more fine-tuned effects specific to meditation. When paired with the study by Williams et al. (2014), our findings suggest that a similar effect is indeed achievable with 36 h less engagement, and future studies should design treatments with different recommendations for practice amount.

The current study raises some doubts about the validity of meditation minutes as a measure of meditation practice dosage. Time spent in the meditation posture does not necessarily equate with time spent in effective practice of meditation, as it also includes time spent in non-target states such as mind wandering. However, there currently exists no clear metric for assessing what constitutes “effective” meditation practice. State measures of mindfulness, such as the Toronto Mindfulness Scale (TMS; Lau et al., 2006) could be used as possible indicators of an effective meditation session and it has been shown to be a predictor of changes in trait mindfulness (Kiken et al., 2015). In addition, some individuals may be more inclined to effectively engage with the practice than others, and therefore more likely to benefit from more time meditating. Future studies need to discover more effective ways of accurately measuring depth or quality of practice (e.g., physiological measures, brain measures, or even self-reported measures of quality that need to be developed in future research).

Future studies should look at moderating effects and participant demographics. The demographics of the participants and instructors in this sample may be of concern as the group was predominantly White, female, and middle-age. Group dynamics may differ in a heterogeneous group or in a different population or cultural context. In the current study, the two participants who reported a significant age gap between themselves and other group members expressed difficulty connecting with other members of the group. Bonding and group cohesion could be reduced or improved based on personal and identity-based characteristics of group members and instructors.

### Conclusion

Common factors research suggests a need to take seriously the therapeutic effect of teachers, participants, and other social and environmental conditions beyond the primary therapeutic modality. The current study found that social common factors – namely the group of other meditators and the instructors – exerted a larger influence on outcomes than the amount of meditation practice. A supportive and trusting alliance with the instructor and with the group may be necessary for participants to be willing to turn toward and stay present with experiences that have been difficult or painful in the past. To the degree that the FFMQ measures a person’s capacity to stay present with, aware of, and non-reactive to the arising of difficult experiences, the results of the current study may suggest that the normalization, validation, and emotional support provided by the group and instructors are essential in terms of encouraging meditators to stay present with and not turn away from their challenging experiences and hence, reduce their negative affect and improve symptoms of stress, anxiety and depression. In this sense, social common factors and mindfulness practice may not be entirely separable as variables, and social common factors may even be more important than the amount of meditation practice itself. As a result, the future success of mindfulness research may depend on its appreciation that MBIs are embedded in a social context.

## Data Availability Statement

The raw data supporting the conclusions of this article will be made available by the authors by request, without undue reservation.

## Ethics Statement

The studies involving human participants were reviewed and approved by the Brown University Institutional Review Board. The patients/participants provided their written informed consent to participate in this study.

## Author Contributions

Co-first authors NC and KE conducted the primary analyses, literature review, and manuscript writing. NC focusing on the quantitative analysis. KE focusing on the qualitative analysis. JL contributed to and advised on the qualitative analysis and manuscript writing. SC contributed to qualitative coding and manuscript writing. JC and WB contributed to and advised on the quantitative analysis and writing the manuscript. WB was the PI of the larger study on dismantling MBCT. All authors contributed to the article and approved the submitted version.

## Conflict of Interest

JC is a Sensei in a Zen Buddhist lineage. WB is a MBSR and MBCT teacher and has received financial compensation for this role. WB is nominally affiliated with the Mindfulness Center at Brown University which generates income by offering mindfulness classes to the public. WB is the founder of Cheetah House, a RI non-profit organization that provides information about meditation-related difficulties, individual consultations, and support groups, as well as educational trainings to meditation teachers, clinicians, educators and mindfulness providers. This interest has been disclosed to and is being managed by Brown University, in accordance with its Conflict of Interest and Conflict of Commitment policies. The remaining authors declare that the research was conducted in the absence of any commercial or financial relationships that could be construed as a potential conflict of interest.
